# Worst pattern of invasion in oral squamous cell carcinoma: An independent prognostic indicator

**DOI:** 10.1016/j.jobcr.2024.12.008

**Published:** 2025-04-10

**Authors:** Manahil Rahat, Umair Aslam Shahzad, Nighat Ara, Hafeez Ud din, Bushra Parveen, Naveed Khan, Saadia Munir, Hassan Mumtaz

**Affiliations:** a Oral & Maxillofacial Pathology, National University of Medical Sciences, Rawalpindi, Pakistan; b Dip RCPath UK; c National University of Medical Sciences Rawalpindi, Pakistan; d Armed Forces Institute of Pathology, Pakistan; e Oral Pathology, National University of Medical Sciences, Rawalpindi, Pakistan; f Data Analytics, BPP University London, UK

**Keywords:** Oral squamous cell carcinoma, Histopathological, TNM staging, Worst pattern of invasion, Lymphovascular invasion

## Abstract

**Introduction:**

Oral squamous cell carcinoma is a malignancy that is biologically aggressive.

**Objective:**

To investigate the correlation between various histopathological factors and the worst patterns of invasion at the tumor-host interface, which were classified as cohesive (1–3) and non-cohesive (4&5).

**Methods:**

Neck dissections were performed on 81 cases of oral squamous cell carcinoma those had been diagnosed. The selection was limited to paraffin-embedded blocks that contained sections from the tumor. Tumor staging, nodal staging and other factors such as lymphovascular invasion, perineural invasion, extra nodal extension, depth of invasion, margin status and tumor differentiation grades were documented.

**Results:**

The findings indicate a higher frequency of non-cohesive worst invasion patterns in numerous anatomical sites. A prediction accuracy of 69.1 % was obtained from the logistic regression analysis, suggesting that the predictive performance has also improved. The chi square test results demonstrated a statistically significant correlation between the variable of interest and extranodal extension showing a p value of 0.008 while lymph node status also showed significant with a p value of 0.000. Another factor that depicted a significance with worst pattern of invasion was tumor margin status having a p value of 0.046. Lymphovascular invasion and the worst pattern of invasion also exhibited a statistically significant correlation, with a p-value of 0.013.

**Conclusion:**

The results of this investigation indicate that aggressive tumor biology is associated with non-cohesive worst pattern of invasion. Non-cohesive worst pattern of invasion is associated with moderate differentiation grade, lymphovascular invasion, perineural invasion, extranodal extension, closed or involved tumor margins and nodal metastases.

## 1 Introduction

Oral cancer is the second most prevalent form of cancer in Pakistan, contributing 10.58 % to the overall disease burden. It is the most prevalent form of cancer among males and accounts for 15.89 % of all reported cases.^1^ Oral squamous cell carcinoma (OSCC) is one of the most frequently diagnosed malignant tumors in Pakistan.^2^ It accounts for over 90 % of oral cancers.^3^ There are numerous indicators that suggest a poor prognosis for OSCC, including advanced tumor stage, higher tumor grade, extra nodal extension (ENE), nodal metastases, greater depth of invasion (DOI), lymphovascular invasion (LVI), poor differentiation, positive surgical margins, and perineural invasion (PNI).^4^

Despite the substantial progress made in the treatment of other forms of cancer, OSCC continues to have a limited number of therapeutic options. Despite the extensive research conducted over the past few decades, the overall five-year survival rate for this illness remains approximately 70 %.^5^ The gold standard for cancer treatment remains surgery, followed by adjuvant radiation therapy and/or chemotherapy. A multimodal approach is necessary for early-stage oral tongue carcinomas that are 4 mm or deeper or have a development pattern of small cell islands or satellites, as they should be treated as high-risk tumors.^6^ Previous investigations into the importance of tumor budding in oral cavity malignancies have associated it with elevated rates of lymph node metastases, relapse, and poor overall survival.^7^

The transition zone between the tumors and the neighbouring stroma is known as the invasive tumor front. A limited number of prior investigations have examined the infiltration pattern of tumors at the tumor front.^8^ This necessitates distinct focus at this time, as The College of American Pathologists has introduced the reporting of oral cavity cancers based on the patterns of tumor invasion. However, these variables are not well-established as prognostic indicators.^9^ This is attributable to the scarcity of published research on invasive oral cavity malignancies.^10^ Despite its limited prognostic significance, the histopathologic grading system endorsed by the World Health Organization (WHO) in its latest edition, OSCC remains straightforward and differentiation based only. It neglects significant variables such as tumor growth pattern and dissociation, stromal reactions (including desmoplasia and local immune response) and tumor-stroma ratio.^11^

The worst pattern of invasion (WPOI) in OSCC refers to the most aggressive and invasive histological pattern observed within the tumor. There is evidence that WPOI increases the likelihood of poor outcomes and locoregional failure. This suggests that WPOI could serve as a predictor of future outcomes and should be taken into account. The classification of various categories of WPOI^12^ is shown in Fig. I.

**Fig. 1:**
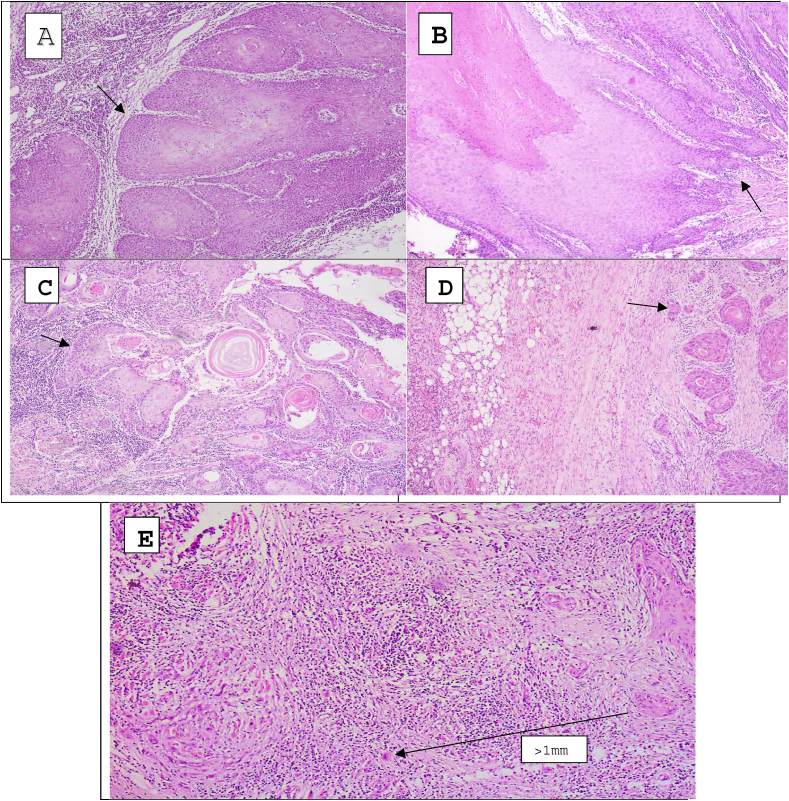
Classification of WPOI in OSCC ^12^ © Photographs Armed forces institute of pathology (AFIP), Pakistan. **A. WPOI Type 1:** Tumor invading in broad pushing manner. **B. WPOI Type 2:** Tumor invading in solid cords and strands (“finger -like"). **C. WPOI Type 3:** Invasive tumor islands with *>*15 cells/cluster. **D. WPOI Type 4:** Invasive tumor islands with *<*15 cells/cluster. **E. WPOI Type 5:** Tumor islands more than 1 mm away from the progressive end of the tumor.

The outcome of OSCC can be predicted by several histological markers that are readily assessed on regular haematoxylin and eosin-stained sections. The metrics that have been mentioned above are excellent indicators of future results. These, in conjunction with the worst pattern of invasion (WPOI) are straightforward and dependable prognostic indicators in early stage OSCC that are associated with a worse prognosis. WPOI assessment is recommended as part of a standardised reporting format for both resection and preoperative biopsy specimens, since it may help with the individualised care of OSCC patients.^8^

## 2 Methods

*Design of study:* The oral pathology department conducted this cross-sectional study from July to November 2023.

*Sample size:* A sample size of 81 was determined using the W.H.O. formula for frequency, as the prevalence of OSCC in the Southeast Asian population is approximately 9.85 %.^13^ Non-probability consecutive sampling was implemented. Eligibility assessment have been showed in Fig. II.

**Fig. 2:**
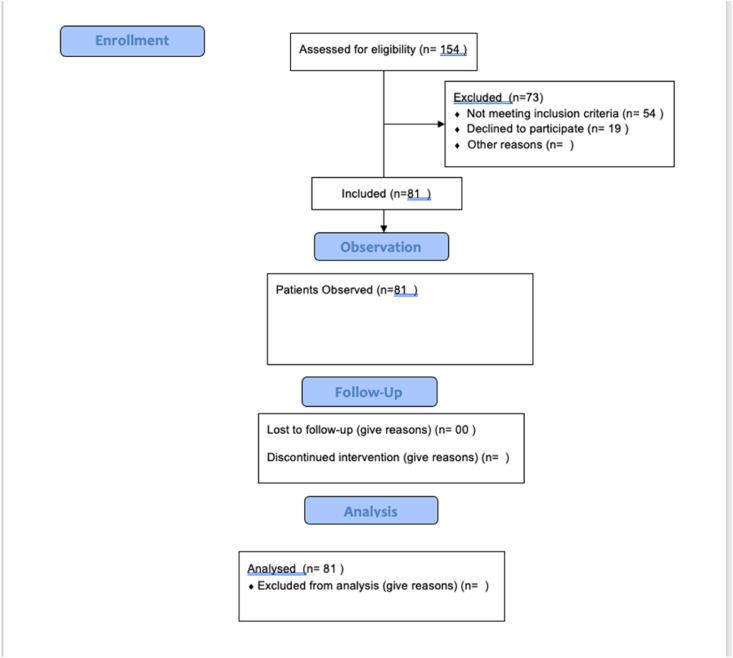
Prisma Chart.

### 2.1 Inclusion criteria

• The investigation encompassed all OSCC patients of both genders and in all age groups who underwent neck dissections.

### 2.2 Exclusion criteria

• All specimens with inadequate fixation.
• All patients' specimen who have undergone chemotherapy and/or radiotherapy prior to surgery.
• Insufficient medical records of patients.

### 2.3 Ethical consideration and data collection procedure

Ethical approval was obtained from the Review Board prior to the commencement of the investigation. Contact information was obtained for all cases of OSCC with neck dissections from the Oral pathology/Histopathology department of Armed forces institute of pathology, Pakistan. By strictly adhering to the exclusion and inclusion criteria, confounding factors were eliminated. The principal investigator of the study initially examined the haematoxylin and eosin slides of these cases. Subsequently, one histopathologist and one oral pathologist independently reviewed and verified the slides to eliminate observer bias. The data collection proforma was employed to document the concluding findings which were subsequently subjected to statistical analysis.

#### 2.3.1 Study variables

Age, gender, tumor site, grade of tumor differentiation (G1:well, G2:moderate and G3:poor), tumor stage (T1, T2 as early stage and T3, T4 as advanced stage disease), lymph node status (according to TNM staging), DOI (<5 mm, 5–10 mm and >10 mm), PNI (seen, not seen), LVI (seen, not seen), ENE (positive, negative), tumor margin status (free >5 mm, close 2–5 mm, involved <1 mm) and worst patterns of invasion (cohesive:1–3, non-cohesive: 4&5).

### 2.4 Statistical analysis

The statistical analysis was conducted using SPSS version 26.0. Diagrammatic representations were created with Microsoft Excel 2019.

To evaluate the significance of the relationship between WPOI with LN involvement, LVI, ENE, T stage, differentiation grades, margin status, DOI, and PNI, the chi-square test was implemented. The p-value of less than or equal to 0.05 was considered statistically significant. In order to ascertain the veracity of the predictions, logistic regression analysis was implemented.

### 2.5 Clinical trial registration

The study was registered at clinical trials.gov under the number NCT 05927220.^14^

## 3 Results

The study consisted of a total of 81 participants, whose ages ranged from 30 to 90 years. The average age of the participants was calculated to be 57.80 ± 12.44 years. Majority of the patients, specifically 71.6 % were male while 28.4 % were female.

Out of total 81 cases of OSCC, 24 were from buccal mucosa (29.6 %), 22 were from lateral border of tongue (27.2 %), 12 from alveolar mucosa (14.8 %), 10 from ventral tongue (12.3 %), 9 from lower lip (11.1 %), 2 from palate (2.5 %) and 2 from upper lip (2.5 %) as shown in Fig. III.

**Fig. 3:**
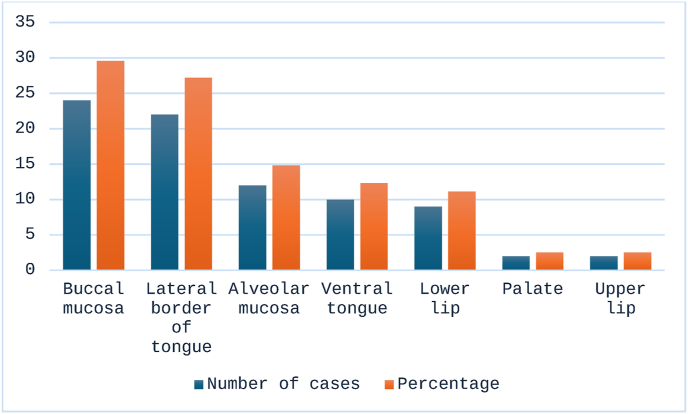
Site Distribution for OSCC cases.

Out of total 81 cases of OSCC, WPOI-4 was seen in the majority number of cases i.e., 36 (44.4 %) as shown in Table I. So, a number of 33 (40.7 %) were cohesive WPOI cases while 48 (59.3 %) were non-cohesive WPOI making non-cohesive WPOI more prevalent as shown in Fig. IV.

**Table 1:** Prevelance of all five types of WPOI in total 81 cases of OSCC.

WPOI	Number of cases	Percentages (%)
**1**	11	13.6
**2**	6	7.4
**3**	16	19.2
**4**	36	44.4
**5**	12	14.8

**Fig. 4:**
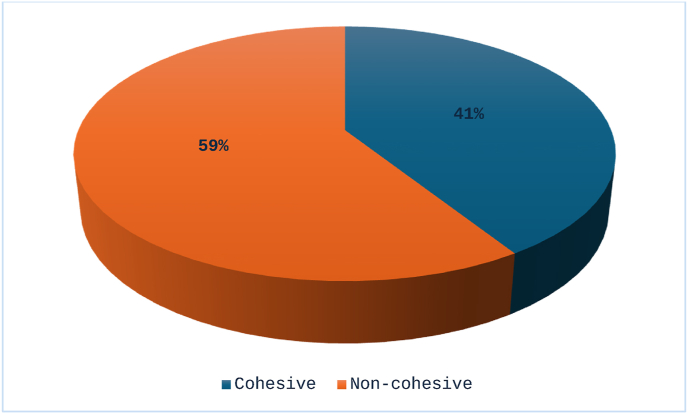
Cohesive (Type 1–3) vs Non-cohesive (Type 4&5) WPOI in OSCC.

The study found a higher frequency of non-cohesive WPOI in specific anatomical areas, with a prevalence rate of 18.5 % in the buccal mucosa and lateral border of the tongue. 9.8 % in the alveolar mucosa whereas, 6.1 % in lower lip. Cohesive WPOI was more common in the palate and upper lip. An equal distribution of both categories was observed in the ventral tongue as shown in Table II.

**Table 2:** Correlation of WPOI with site of tumor.

Anatomical sites	WPOI	
	Cohesive	Non- cohesive
Buccal mucosa	9 (11.1 %)	15 (18.5 %)
Lateral border of the tongue	7 (8.6 %)	15 (18.5 %)
Alveolar mucosa	4 (4.9 %)	8 (9.8 %)
Ventral tongue	5 (6.1 %)	5 (6.1 %)
Lower lip	4 (4.9 %)	5 (6.1 %)
Upper lip	2 (2.4 %)	0
Palate	2 (2.4 %)	0

Table III indicates that 17 of the 33 cases with cohesive WPOI exhibited Grade 1 tumor differentiation, 15 exhibited Grade 2, and only 1case was Grade 3. While 18 of the 48 non-cohesive WPOI cases were classified as Grade 1, 25 as Grade 2, and 5 as Grade 3 tumors.

**Table 3:** WPOI Association with Grade of Tumor differentiation.

WPOI		Tumor Differentiation		P-Value
	G1	G2	G3	
**Cohesive**	17	15	1	0.286
**Non-Cohesive**	18	25	5	

According to Table IV, out of the 33 cases of cohesive WPOI, 25 cases exhibited early stage tumors, while 8 cases exhibited advanced stage tumors. From the total 48 non-cohesive WPOI cases 27 were early-stage and 21 were advanced stage tumors.

**Table 4:** WPOI association with tumor size.

WPOI	Tumor Size		p-Value
	Early Stage	Advanced Stage	
**Cohesive**	25	8	0.068
**Non-Cohesive**	27	21	

Extranodal extension (ENE) was present in 9 (11.1 %) of non-cohesive WPOI cases while none in cohesive WPOI cases as shown in Table V.

**Table 5:** Association of ENE with WPOI cases in OSCC.

WPOI	EXTRANODAL EXTENSION (ENE)		Total	p-value
	Positive	Negative		
**Cohesive**	0	33	33	0.008
**Non-cohesive**	9	39	48	
**Total**	9	72	81	

Similarly, Lympho vascular invasion (LVI) also showed a significant association with WPOI (p = 0.013). The last histopathological factor that showed significant association was lymph node status (p = 0.000), as shown in Table VI.

**Table 6:** Relation of Lympho-vascular invasion (LVI) & Lymph node status with WPOI in OSCC.

**WPOI**	**LYMPHOVASCULAR INVASION (LVI)**			**p-Value**
	**Not seen**	**Seen**	**Total**	
**Cohesive**	33	0	33	0.013
**Non-cohesive**	40	8	48	
**Total**	73	8	81	
**WPOI**				
**LYMPH NODE STATUS**	**Cohesive**	**Non-cohesive**	**Total**	

**Involved**	4	28	32	0.000
**Uninvolved**	29	20	49	
**Total**	33	48	81	

A significance between WPOI and margin status was also seen having a p value of 0.046 as shown below in Table VII.

**Table 7:** Relation of margin status with WPOI in OSCC.

WPOI	Margin Status			p value
	Close	Free	Involved	
**Cohesive**	15	17	1	0.046
**Non cohesive**	18	19	11	

Logistic regression analysis showed improved prediction results, with 30 cases correctly predicted as cohesive and 3 cases correctly predicted as non-cohesive resulting in an overall percentage of correct predictions of 69.1 %, as shown in Table VIII.

**Table 8:** Logistic regression analysis.

Observed	Prediction		Percentage
WPOI	WPOI		
	Cohesive	Non-cohesive	
**Cohesive**	30	3	90.9
**Non-cohesive**	22	26	54.2
**Overall Percentage**	52	29	69.1

## 4 Discussion

There has been a significant global escalation in the incidence, prevalence, and fatality rates associated with oral cancer.^15^ A study conducted in Lahore, Pakistan on 86 participants showed 75.58 % (n = 65) of the male participants and 24.42 % (n = 21) female participants who were affected by OSCC.^16^ Similarly, a study conducted in India showed a male-to-female ratio of 3:1 for development of this form of cancer.^17^ The prevailing trend has also been seen in the current investigation where it was found out that 71.6 % (n = 58) males and 28.4 % (n = 23) females, as in the majority of studies undertaken on patients with OSCC in Pakistan, indicating a higher proportion of male patients. One possible explanation for this phenomenon could be because males have a higher prevalence of engaging in unlawful habits such as snuff dipping and smoking, in comparison to females.

The age range for the participants was 30–90 years old and the mean age of patients was found to be 57.80 ± 12.44 years in present study. The findings of one study revealed that the age group with the largest percentage of patients diagnosed with OSCC was 51–60 years old. This was followed by the age groups of 41–50 and 61–70. The average age of the entire population was 51.09 ± 14.36 years which are almost consistent with results of this study.^18^ The data set from another study conducted over a ten-year duration in Iran showed a mean of 59.3 ± 15.7 years. The age groups comprised individuals in their seventh and eighth decades with the highest occurrence of disease, showing similarity with results of this study.^19^

The most common site for developing OSCC in this study was buccal mucosa 29.6 % (n = 24) followed by lateral border of tongue 27.2 % (n = 22) which is consistent with a study conducted in Pakistan on a population size of 186 individuals that showed frequency of OSCC in the buccal mucosa to be twice as high in individuals who chew tobacco. This association was particularly apparent among males and those with a lower socioeconomic status. These findings are consistent with many regional studies that have identified the buccal mucosa as the most often affected site for OSCC.^20^

The utilization of the TNM system for clinical assessment is a common and established practice in determining the magnitude of tumor burden and subsequently selecting appropriate treatment strategies for individuals diagnosed with OSCC. A prominent critique of the TNM method is its disregard for the specific histological attributes of tumors. The examination of tumor behaviour can be conveniently assessed by the observation on routine H&E histopathology preparations. In order to facilitate this process, numerous histological characteristics have been examined and duly considered in the determination of adjuvant therapy strategies. One such characteristic indicator is WPOI which is described as the invasive front of the tumor at the tumor-host interface.^12^

The main objective of this research was to evaluate and determine a relationship between the different kinds of histopathological variables and cohesive (1–3) and non-cohesive (4&5) WPOI at the tumor-host interface in cases with OSCC. It was found out that most of the cases demonstrating WPOI-4 with a total of 36/81(44.4 %) cases and broadly 48/81 (59.3 %) cases with non-cohesive WPOI (4&5). This is concurrent with the findings of Mishra et al.^12^ which also showed WPOI type 4 as the most common pattern.

Malignant cells show increased inclination to undergo metastasis. The findings in current study align with the observation that there is a correlation between non-cohesive WPOI (4&5) and an increased occurrence of lymph node metastases (p < 0.05). Another study displayed that Worst pattern of tumor invasion (type 4 and 5) are associated with higher risk of LN metastasis.^8^

A significant association between ENE and non-cohesive WPOI (4&5) OSCC cases in this study (p = 0.008) was seen. This was concurrent with multiple studies carried out at different times collectively stated in a meta-analysis comprehensively done by Dolens et al.^10^

This study indicates non-cohesive WPOI (4&5) to be more prevalent in early-stage tumor (pT1 and pT2) showing 25 (30.8 %) for cohesive and 27 (33.3 %) for non-cohesive cases. This is against the findings with the study conducted by Mishra et al.^12^ in which type 4 and 5 WPOI are associated with larger tumor sizes.

The classification of OSCC into well, moderate, and poorly differentiated categories is determined by evaluating the level of keratinization and cytological maturation observed in histological analysis. The predictive value of this rating is limited. Nevertheless, there is a discernible pattern indicating a propensity for regional lymph node metastasis, with higher grade tumors exhibiting a notable escalation in nodal metastasis.^21^ The results of this study showed 30.8 % moderately differentiated OSCC exhibiting non-cohesive WPOI having similarity with study conducted by Mishra et al.^12^ which displayed majority of cases with WPOI-4 as moderately differentiated OSCC.

The findings of this study indicate that a significant proportion of patients with involved and closed margins were related with WPOI type 4. This observation is consistent with previous researches conducted by Almangush et al., Rodrigues et al.^11^^,^^22^ and Mishra et al.^12^ Also, this study found a significance between WPOI and margin status (p = 0.046). This observation demonstrates that in cases of non-cohesive WPOI, tumor has the ability to extend beyond the clinical borders. It is reasonable to consider incorporating broader margins of excision during surgical procedures for instances that have pathologically determined in pre-operative biopsy. While the potential impact on survival remains uncertain, it is plausible that this intervention could lead to a decrease in the incidence of local recurrence.^23^

Depth of invasion reflects the tumor progressiveness. As the DOI increases above 5 mm, there is a corresponding increase in the likelihood of developing WPOI 4 and 5.^24^ Superficial tumors have lower levels of aggressiveness in comparison to tumors with a higher degree of invasiveness. It should be noted that a number of studies have demonstrated enhanced survival results by the incorporation of adjuvant therapy informed by the depth of invasion (DOI). This observation suggests a potential hypothesis that individuals exhibiting WPOI 4 & 5 may experience potential benefits from therapy intensification.^12^

A study conducted by Lundqvist in 2011 interpreted that tumor exhibiting invasive fronts have a propensity to disseminate by perineural invasion, which is considered a physiologically aggressive characteristic in OSCC.^25^ However only 5 cases with non-cohesive WPOI were seen to have PNI positivity in this study, hence showed no significance which is contradictory to previous studies conducted by Batool et al.,^26^ Mishra et al.^12^ and Marzouki et al.^27^

Significantly, Lymphovascular invasion (LVI) is recognized as a pathological occurrence wherein tumor cells infiltrate a vascular or lymphatic region that is lined by endothelium, without invading the underlying muscles. The infiltration of tumor cells into lymph vascular spaces via traversing the endothelial cell layer is regarded as a crucial stage in the progression of tumor metastasis. This phenomenon has been identified as a noteworthy prognostic characteristic in various types of cancer, including prostate cancer and colorectal cancer.^28^ The study by Huang et al.^28^ showed a significant association between LVI and LN metastasis demonstrating a poorer prognosis in patients with LVI positivity. Similarly, LVI was found to be significant in non-cohesive WPOI cases (p = 0.013) in this study.

Vascular invasion constitutes a significant risk factor for the occurrence of regional nodal metastasis and is associated with an unfavorable outcome. So, it is advisable to consider elective neck dissection in patients with clinical stage I and II when perineural/vascular invasion is detected.^29^

### 4.1 Limitations of the study

Single centre Study: This study was conducted at a single centre, perhaps limiting its ability to properly capture the range of patients and treatment modalities found in other clinical settings. Conducting investigations at multiple centres could offer a more thorough comprehension of the phenomena being investigated.

Histopathological Variation: Histopathological analysis is prone to subjectivity and might differ across pathologists. The diversity in grading WPOI especially between type 4 and 5 among observers may impact the consistency and reliability of the findings.

### 4.2 Call for further research

The study emphasizes the necessity of conducting new research using consistent approaches in order to address the deficiencies in the current knowledge. In order to improve our understanding of the predictive significance of WPOI as a histopathological marker in OSCC, future research should focus on increasing sample numbers, including varied populations, and utilizing prospective study approaches.

## 5 Conclusion

This study validates the potential prognostic value of numerous histopathological characteristics and emphasize the encouraging outcomes of others. This study is one of its kind where role of WPOI as a separate prognostic marker has been evaluated in oral squamous cell carcinoma. While there are 5 patterns noted, distinction between WPOI-4&5 (non-cohesive types) and other cohesive patterns i.e., type 1–3 is what is most relevant. This study discovered that a significant proportion of cases of OSCC demonstrated non-cohesive WPOI and majority of cases exhibited WPOI-4.

This study has found that non-cohesive WPOI is associated with aggressive tumor biology. The presence of WPOI type 4&5 is associated with moderate tumor differentiation, involved or closed tumor margins, PNI, LVI, ENE and higher incidence of nodal metastasis, as observed in this investigation. The depth of invasion is indicative of the tumor's level of progression. As the DOI exceeds 5 mm, there is a proportional rise in the probability of developing WPOI type 4&5.

Patients with non-cohesive WPOI have a poorer oncological outcome in terms of disease-free survival and overall survival irrespective of stage and adjuvant treatment due to aggressiveness associated with these patterns. The results of this study show, patients with non-cohesive WPOI (4&5) may be considered for treatment intensification especially in early-stage tumors. This may lead to improved survival rates.

Nevertheless, it is recommended to conduct additional studies with consistent methodologies to address the existing gaps in the literature regarding the relevance of histopathological markers for predicting the prognosis of OSCC.

## Sources of funding

No funding received.

## Declaration of competing interest

The authors declare that they have no known competing financial interests or personal relationships that could have appeared to influence the work reported in this paper.
